# The use of adherence aids by adults with diabetes: A cross-sectional survey

**DOI:** 10.1186/1471-2296-7-1

**Published:** 2006-01-05

**Authors:** Benjamin Littenberg, Charles D MacLean, Laurie Hurowitz

**Affiliations:** 1 University of Vermont, General Internal Medicine, 371 Pearl Street, Burlington, Vermont, 05401, USA

## Abstract

**Background:**

Adherence with medication taking is a major barrier to physiologic control in diabetes and many strategies for improving adherence are in use. We sought to describe the use of mnemonic devices and other adherence aids by adults with diabetes and to investigate their association with control of hyperglycemia, hyperlipidemia and hypertension.

**Methods:**

Cross sectional survey of diabetic adults randomly selected from Primary Care practices in the Vermont Diabetes Information System. We used linear regression to examine the associations between the use of various aids and physiologic control among subjects who used oral agents for hyperglycemia, hypercholesterolemia, and hypertension.

**Results:**

289 subjects (mean age 65.4 years; 51% female) used medications for all three conditions. Adherence aids were reported by 80%. The most popular were day-of-the-week pill boxes (50%), putting the pills in a special place (41%), and associating pill taking with a daily event such as a meal, TV show, or bedtime (11%). After adjusting for age, sex, marital status, income, and education, those who used a special place had better glycemic control (A1C -0.36%; *P *= .04) and systolic blood pressure (-5.9 mm Hg; *P *= .05) than those who used no aids. Those who used a daily event had better A1C (-0.56%; *P *= .01) than patients who used no aids.

**Conclusion:**

Although adherence aids are in common use among adults with diabetes, there is little evidence that they are efficacious. In this study, we found a few statistically significant associations with adherence aids and better diabetes control. However, these findings could be attributed to multiple comparisons or unmeasured confounders. Until more rigorous evaluations are available, it seems reasonable to recommend keeping medicines in a special place for diabetic adults prescribed multiple medications.

## Background

Adherence has long been recognized to be a major barrier to optimum care of chronic diseases, including diabetes [1-3]. Typical rates of medication adherence are about 50%, with a very wide range [4,5]. Many approaches have been offered to improve adherence including patient education, written information, behavior modification, cash incentives, directly observed therapy, and even incarceration [6-9]. Although medication-taking behavior has many components, forgetfulness has been noted to be a major mechanism of non-adherence, especially in chronic disease [3,10]. A number of maneuvers have been proposed to enhance adherence, often by compensating for poor memory [11]. These aids range from simple paper-and-pencil checklists and calendars [12], to special packaging with calendar blister packs, to sophisticated computerized dispensing machines that pop open a small drawer containing pills and signal the patient when a dose is due. One of the earliest devices (patented in 1894) was a printed clock face that was attached to medication bottles to indicate when the next dose was due [13]. Recently, home computers, e-mail, and personal paging systems have appeared [14,15].

Data on the prevalence of adherence aids in clinical practice are unavailable for any condition, including diabetes. Likewise, the efficacy of most of these devices is unstudied. Calendar blister packs have been studied for infectious diseases and hypertension, (with mixed results [16,17]), but not, apparently, for diabetes. A variety of educational and support interventions have been studied for several other chronic conditions [18]. Again, the results have been mixed and diabetes has not been not the target condition.

Even modest increments in adherence could have substantial impacts upon clinical outcomes and costs [19-21]. Adherence aids might not have to be widely successful to be valuable. For instance, an intervention such as a day-of-the-week pill box or a medication calendar, that costs no more than a few dollars per patient, would be cost-effective if it helped just one patient in hundreds lower their A1C by just one percentage point. We are unaware of any systematic studies of either the prevalence or effectiveness of adherence aids in diabetes.

## Methods

As part of a larger study of diabetes, the Vermont Diabetes Information System [22], we recruited 125 Primary Care providers from 69 practices across Vermont, New Hampshire, and upstate New York For this analysis, we randomly selected adults with diabetes (confirmed by the provider) from each practice. Patients whose provider noted them to be severely demented or residing in a nursing home were eliminated. After receiving an introductory letter from their provider, potential subjects were telephoned and invited to participate in an interview at their home.

Subjects who agreed were mailed a questionnaire and were scheduled for an interview by a trained field interviewer. The questionnaire covered multiple domains including demographics, medication usage, and use of adherence aids. The adherence aid items included a list of known aids and asked the patients to record all that they used. During the visit, the interviewer reviewed any missing or ambiguous questionnaire items. If necessary, the interviewer read the questions aloud for subjects and recorded their responses for them. Then the interviewer measured the subjects' height and weight and blood pressure (three times at five minute intervals). The subjects were asked to produce every medicine (including prescription, non-prescription, and over-the-counter medications, herbal preparations, vitamins, and supplements) they had used in the past month for the interviewer to record.

The local community hospital clinical laboratories provided the most recent measure of glycosolated hemoglobin (A1C) and LDL-cholesterol for each subject.

We used basic descriptive statistics to describe the proportion of subjects reporting use of the various aids. Blood pressure was analyzed as the mean of the three recorded systolic pressures. We used chi-square tests to compare proportions and t-tests to compare means. For aids reported by at least 10% of subjects, we calculated the levels of physiologic control (mean A1C, mean LDL-cholesterol, and mean systolic blood pressure) for those using and not using the aid. The association of physiologic control with aid usage was evaluated with least-squares linear regression. Because social and economic factors may be associated with both adherence aid use and physiologic control, we adjusted for potential confounders by expanding the regression models to include covariates for patient age, sex, income (in seven ordinal categories), education (in seven ordinal categories), race ("white" or not) and marital status ("currently married or living as such" or not). We restricted the analysis to subjects who used oral medications from all three classes under consideration and had complete data on adherence use, physiologic control, and socioeconomic status. Subjects who were using only insulin without an oral hypoglycemic agent were excluded.

The study protocol was approved by the University of Vermont Committee for the Protection of Human Subjects (CHRMS# 01–211) and was performed in full compliance with the Helsinki Declaration. All interviewed subjects provided fully informed consent.

## Results

71% of contacted subjects agreed to the field survey. Of 1,007 interviewed subjects, 136 were interviewed before the adherence aide questions were included in the survey, 526 were not using oral medications from all three classes, and 56 had incomplete data. The average age of the 289 remaining subjects was 65.4 years with a range from 33 to 90 years; 51% were female. Seventy-two percent were high school graduates; 16% finished at least a four year college degree. Ninety-eight percent were white. The mean A1C at the last measured value before the interview was 7.2%. 52% were under control (A1C <7.0%). The mean LDL was 96 mg/dl; 57% were below the target of 100 mg/dl. The mean blood pressure was 143/78 mm Hg with 20% at 130/80 mm Hg or lower. The mean body mass index was 34.5 kg/m^2 ^with 71% in the obese range (30 – 61 kg/m^2^).

All 289 subjects were using at least one oral agent for each of hypertension, hypercholesterolemia, and hyperglycemia. The average number of medications (including as-needed preparations) was 10.5 with a range from 3 to 29. Prescription medications accounted for 8.3 of these while non-prescription preparations averaged 2.2 per subject. Insulin was used by 16% of respondents.

The included subjects were similar to those not taking medications from all three classes in terms of age, sex, race, income, and education. As expected, they used more medications (10.5 vs. 7.5; *P *< 0.001), and had higher A1C (7.2 vs. 7.1%; *P *= 0.01), LDL-cholesterol (107 vs. 96 mg/dl; *P *< 0.001), and systolic blood pressure measurements (143 vs. 140 mm Hg; *P *= 0.07). Body mass index was also higher in the included subjects (34.4 *vs*. 33.2 kg/m^2^; *P *= 0.01)

Two-hundred-thirty-one subjects (80%) reported using one or more aids. One-hundred-thirty-two (46%) reported one aid; 61 (21%) reported two aids, 29 (10%) reported three aids; nine (3%) reported four or more aids. The most popular aids were the day-of-the-week pill box (50% of all respondents), keeping medicines in a special place (41%), and associating medicines with a daily event such as a TV show or meal (17%). See Table 1.

**Table 1:** Use of various adherence aids in 289 adults with diabetes

Adherence Aid	Number	%
I use a day-of-the-week pill box	143	49.5
I put my pills in a special place that reminds me	117	40.5
A daily event (a meal, TV show, bedtime, brushing my teeth) reminds me	50	17.3
My family or friends remind me	27	9.3
I check off my medicines on a list	12	4.2
Someone gives me my medicines each time	10	3.5
I move the medicines from one place to another	9	3.1
I use an electronic pill box or dispenser	4	1.4
I mark a calendar or diary when I take my medicines	3	1.0
The pharmacy makes special packets of pills for each time I take my medicine	3	1.0
I set a timer	3	1.0
I leave a note for myself	3	1.0
My pager beeps to remind me	2	0.7
I move my watch or jewelry from one hand to the other	1	0.4
I receive a phone call to remind me	0	0
**Any one or more of the above**	231	79.9

37% of subjects reported using more than one adherence aid.

Among subjects who reported that "I put my pills in a special place that reminds me," compared to those who used no adherence aides, physiologic control was somewhat better in all three areas: glycemic control (A1C = 7.05 *vs*. 7.44%; *P *= 0.03), systolic blood pressure (139.6 *vs*. 144.7 mmHg; *P *= 0.08), and LDL-cholesterol (93.8 *vs*. 94.4 mm/dl; *P *= 0.90). After adjusting for possible social and economic confounders, glycemic control and blood pressure, but not hypercholesterolemia, were significantly better in those who used the aide. See Table 2.

**Table 2:** Multivariate associations between adherence aid use and physiologic control in 289 adults with diabetes

Aid	A1C		Systolic Blood Pressure		LDL-cholesterol	
	*%*	*P*	*mm Hg*	*P*	*mg/dl*	*P*
Special place	**-0.36**	**.04**	**-5.9**	**.05**	+0.4	.94
Daily event	**-0.56**	**.01**	+1.5	.73	+0.6	.92
Pill box	-0.11	.57	-2.9	.35	+7.2	.16
Any aid	-0.24	.15	-3.5	.24	+3.0	.52

Linear regressions between adherence aid use and physiologic control adjusting for age, sex marital status, income, race, and education. A negative sign indicates that use of the aid was associated with a lower (better) value of the physiologic measure.

Among those who reported that "A daily event (a meal, TV show, bedtime, brushing my teeth) reminds me," glycemic control was significantly better than those who used no aides (A1C = 6.87 *vs*. 7.44%; *P *= 0.005). However, the other physiologic domains were not significantly different: systolic blood pressure (143.4 *vs*. 144.7 mmHg; *P *= 0.72), and LDL-cholesterol (95.8 *vs*. 94.4 mm/dl; *P *= 0.81). The benefit for glycemic control and lack of efficacy for blood pressure and cholesterol were confirmed in the multivariate analyses. See Table 2.

About half of subjects indicated that "I use a day-of-the-week pill box." Compared to those who used no aids, there was no significant association with glycemic control (A1C = 7.19 *vs*. 7.44%; *P *= 0.16), systolic blood pressure (141.9 *vs*. 144.7 mmHg; *P *= 0.35) or LDL-cholesterol levels (101.2 *vs*. 94.4 mm/dl; *P *= 0.17). Again, this pattern of effect was not altered by multivariate adjustment for possible confounders (Table 2).

Users of any one or more aids had a mean A1C of 7.13%. Among the non-users, the mean A1C was 7.44% (*P *= 0.05). There was no significant advantage for aid users in mean LDL level (97.0 *vs*. 94.4 mg/dl; *P *= 0.57) or systolic blood pressure (142.0 *vs*. 144.7 mmHg; *P *= 0.37). In multivariate analyses, none of the physiologic risk factors were significantly different between users and non-users. See Table 2.

## Discussion

A wide variety of adherence aids are in common use among adults with diabetes. More than three quarters of these subjects use one or more aids, with half using a day-of-the-week pill box. A few aides were associated with better physiologic control and these findings persisted when adjusting for social and economic factors.

However, several limitations of this analysis should be kept in mind. Subjects were not randomly assigned to the use of aids. Therefore, the relationship between use of adherence aids and physiologic control may be confounded by unmeasured variables. In other words, the patients who use aids may have other behaviors or characteristics that influence their laboratory results. For instance, it is possible that patients who use mnemonic devices for their medicines also tend to exercise and follow their diet.

We performed three regression analyses on each of four aides, raising the possibility of falsely declaring statistical significance when no association exists (Type I error due to multiple comparisons). If we raise the threshold for significance with the Bonferroni correction to 0.05/12 = 0.0042 [23], none of the analyses demonstrate statistical significance.

Patients with diabetes receiving care in mostly rural primary care practices were randomly invited to participate in the study. Our results may not generalize to populations from other settings. In particular, this population is primarily older adults with Type 2 diabetes using multiple prescription medications. These results may not generalizable to diabetic patients on simpler regimens or patients with other chronic conditions. Further, it is possible that patients who consented to the interview are systematically different than refusers in regard to their use of adherence aids.

## Conclusion

Medication adherence remains an important barrier to optimum patient care [24]. Although adherence aids are in common use among adults with diabetes, there is little evidence that they are efficacious. In this study, a few associations with better control were observed, but they may be artifacts of multiple comparisons or unmeasured confounders. Until more rigorous evaluations are available, it seems reasonable to recommend keeping medicines in a special place for diabetic adults prescribed multiple medications.

## Competing interests

The author(s) declare that they have no competing interests.

## Authors' contributions

BL conceptualized the study, performed the analysis, and drafted the manuscript. CDM contributed to the design and analysis, supervised the data collection, and edited the manuscript. LH made major analytical and design contributions and edited the manuscript. All authors read and approved the final manuscript.

## Pre-publication history

The pre-publication history for this paper can be accessed here:


